# Structural equation modelling of food safety knowledge, attitude and practices among consumers in Malaysia

**DOI:** 10.1371/journal.pone.0235870

**Published:** 2020-07-08

**Authors:** Jan Mei Soon, Ikarastika Rahayu Abdul Wahab, Ruhil Hayati Hamdan, Mohd Hafiz Jamaludin

**Affiliations:** 1 Faculty of Health and Wellbeing, University of Central Lancashire, Preston, England, United Kingdom; 2 Faculty of Agro-Based Industry, Universiti Malaysia Kelantan, Jeli, Kelantan, Malaysia; 3 Faculty of Veterinary Medicine, Universiti Malaysia Kelantan, Kota Bharu, Kelantan, Malaysia; Univerza v Mariboru, SLOVENIA

## Abstract

Factors contributing to foodborne illnesses in Malaysia were identified as insanitary food handling procedures and lack of hygiene in food preparation area. Food safety at home is a critical point as consumers represent the final step in food preparation and prevention of foodborne diseases. This study aims to investigate the food safety knowledge, attitude and self-reported practices among consumers in Malaysia. An online survey was conducted, and data were analysed using descriptive statistics and exploratory factor analysis. A model linking food safety knowledge and attitude and their direct effects on practices were confirmed using structural equation modelling (SEM). The proposed model fulfilled the goodness of fit indices and is deemed acceptable. Respondents demonstrate good level of food safety knowledge and positive attitudes and self-reported practices. Food safety knowledge has a negative and insignificant relationship with food safety practices (β1 = -0.284, p>0.05) while attitude significantly affects food safety practices (β1 = 0.534, p<0.05). The findings clearly indicate that food safety knowledge does not directly affect food safety practices This is also the first study to provide new empirical findings on thermometer usage among consumers in Malaysia. This study establishes an important point of reference where consumers use visual appearances to determine if food is thoroughly cooked and practice washing raw chicken prior to cooking. Food safety practices at home play a critical role in protecting consumers in reducing risks of foodborne illnesses.

## Introduction

The World Health Organization estimated that foodborne diseases caused 600 million cases and 420,000 deaths annually of which 30% of the fatalities occur among children under 5 years of age [1]. The main contributing factor to foodborne diseases in Malaysia was identified as insanitary food handling procedures and lack of cleanliness in premises which accounted for more than 50% of the poisoning incidents [2]. Most of the reported outbreaks were recorded at educational institutions and schools. This has led to an increase of food safety studies on knowledge, attitude and practices among commercial food handlers in Malaysia [3–7]. These studies consistently revealed that food handlers were unaware of different foodborne pathogens and had poor to average knowledge of hygienic practices in food handling. [8] revealed that although the food handlers in their study exhibited excellent level of food safety knowledge and attitude, they could further improve on hygiene practices such as cleaning the food preparation and working area and to avoid wearing jewelleries whilst working. Although numerous studies had been conducted among food handlers at food service operations, food safety at home is another critical point as consumers represent the final step in food preparation and prevention of foodborne diseases. More than 35% of foodborne diseases occurred at home [9]. This represents the tip of the iceberg as the number of reported cases are underestimated due to the lack of outbreak reports in home settings [10, 11]. Consumers who misinterpret foodborne disease symptoms or treat food poisoning symptoms as transient inconveniences represent the reasons why consumers do not often seek medical treatment [9, 11, 12].

The evaluation of food safety among Malaysian consumers had been conducted although the studies were mostly conducted in East Malaysia [12–14]. [14] discovered that all the Theory of Planned Behaviour antecedents i.e. food safety knowledge, subjective norm and perceived behavioural control showed significant effects on the intention of safe food handling. One of the key predictors of intention to safe food handling were subjective norm, demonstrating that encouragement from family members could improve food safety practices at home. [12] evaluated the consumers’ knowledge of hygienic practices and revealed that consumers demonstrated poor attitude in the use of cutting boards and used the floor as cutting board instead. [13] further assessed consumers’ food safety knowledge of personal hygiene, foodborne disease symptoms, high risk foods, cross contamination and temperature control. The overall food safety knowledge of adult consumers was considered good but was seriously lacking in knowledge of temperature control. Similarly, a survey on microwave oven safety revealed low level of microwave oven knowledge and safety practices [15]. More than 70% of the respondents did not reheat food periodically nor stir their food midway of the reheating process. This could potentially lead to non-uniform heating leading to presence of cold spots which will allow bacteria (if present) to survive and grow when conditions are optimal. Knowledge of temperature control remain poor among consumers and food handlers. This is supported by an intervention study conducted by [16] where food safety training failed to increase knowledge of temperature control such as awareness of the temperature danger zone and the correct temperatures for food storage and heating.

Temperature control and thermometer usage remain a challenge in Malaysia, not only among commercial food handlers but even more so among food handlers at home. The hot and humid climate of this country contributes to the optimal growth of most mesophilic foodborne pathogens [17]. To what extent do consumers use thermometers at home? What are the reasons for not using a thermometer? There is also a lack of food safety studies among consumers in Peninsular Malaysia. This study investigates the food safety knowledge, attitude towards food safety and food safety practices among general consumers. Food safety knowledge refers to the understanding of or information about food acquired through experience or education while attitude is a feeling or opinion about food safety and practice refers to the action or application of food safety. Structural equation modelling (SEM) had been used in a number of food safety studies [12, 18–21]. In this study, the author postulates the following hypotheses based on [12, 18, 20]. This study proposed the following hypotheses:

H1: Food safety knowledge does not directly affect food safety practices
H2: Attitude towards food safety directly affects food safety practices
H3: Food safety knowledge and attitude are correlated

This study utilises SEM as a confirmatory technique to determine the proposed model validity and to examine the relationship between food safety knowledge, attitude and practices among consumers in Malaysia.

## Methodology

### Questionnaire development

The questionnaire was constructed based on [12, 18, 22–23] and was divided into 5 sections: (i) demographics; (ii) thermometer usage; (iii) knowledge; (iv) attitudes and (v) practices. The surveys conducted in previous studies were based on recommended food safety handling practices and fulfilled the WHO Five Keys to Safer Food practices [23] i.e. keep clean; separate raw and cooked; cook thoroughly; keep food at safe temperatures; and use safe water and raw materials. In the food safety knowledge section, participants were provided with optional answers i.e. ‘yes’, ‘no’ and ‘uncertain’ to prevent participants from selecting the correct answer by chance. The food safety attitude and practices questions allow participants to rate on a 5-point Likert scale of strongly disagree/never to strongly agree/always. The questionnaire was provided in both English and Malay languages. The questions were translated into Malay and back translated into English to ensure accuracy. A pilot study was conducted among 15 participants at a local university to evaluate the language, clarity and suitability of wordings. The pilot data were not included in the final analysis. A copy of the questionnaire in both Malay and English is provided in Supporting Information (S1 Questionnaire).

### Data collection

Ethical approval was obtained and granted by University of Central Lancashire HEALTH Ethics committee (0009). The questionnaire was uploaded onto Online Survey (previously Bristol Online Survey) and sends an online survey announcement to its sample pool and recruit respondents over the age of 18 for this study. The inclusion criteria for the participants are that they should be adult Malaysia residents and are currently residing in Malaysia. Participant consent was obtained before the start of the survey and all participants could withdraw from the survey by simply exiting or closing the browser page. Survey is a useful tool to obtain a high volume of information from a large number of people in a short period of time [24]. The online survey was conducted between June–September 2019. A reminder was sent to all potential respondents in August 2019 to boost the number of responses. All returned responses were checked and verified by the authors to ensure completion. A total of 793 questionnaires were returned of which 787 surveys were valid. Fully completed surveys were deemed as valid responses.

### Statistical analysis

Descriptive and Exploratory Factor Analysis (EFA) was conducted using Statistical Package for Social Science (SPSS) 26.0 software. EFA was performed to extract valid items for knowledge, attitude and practices. This is based on factor loadings of the scale items greater than 0.40 (Baser et al., 2017). Confirmatory Factor Analysis (CFA) was performed using Analysis of Moment Structures (AMOS) and confidence level was set at 95%.

## Results

Table 1 shows the demographic characteristics of the participants. Up to 2/3 of the respondents are female and 87.0% are below the age of 36 and more than 90% are currently studying at institutions of higher education or had received tertiary education. Up to 75.7% of the respondents reported having experienced symptoms of food poisoning with diarrhoea (71.2%) and vomiting (56.4%) being the most common symptoms. Although 85.1% of respondents prepared meals at home, only 15.1% reported using a thermometer. Amongst those who reported using a thermometer, 91.6% of them uses the thermometer correctly. The main reasons for not using a thermometer was that the respondents knew the food is cooked by checking its visual appearance (53.3%), troublesome to use it (15.3%) and do not know how to use it (10.9%).

**Table 1:** Demographic characteristics of participants (n = 787).

Variable	Items	Frequency (%)
Gender	Male	222 (28.2)
	Female	565 (71.8)
Age	18–25	294 (37.4)
	26–35	390 (49.6)
	36–45	69 (8.8)
	46–55	29 (3.7)
	≥ 56	5 (0.6)
Education	Primary	4 (0.5)
	Secondary	61 (7.8)
	Tertiary	722 (91.7)
Have you experienced food poisoning before	Yes	596 (75.7)
	No	127 (16.1)
	Uncertain	64 (8.1)
What were the symptoms experienced? Please tick any that applies:	Nausea	307
	Vomiting	444
	Diarrhoea	560
	Stomach cramps	443
	Fever	188
	Tired	354
	Aches	158
	Chills	127
	Headaches	160
	Loss of appetite	321
	Others	4
Do you prepare your own or for family meals?	Yes	307 (39.0)
	No	117 (14.9)
	Sometimes	363 (46.1)
If you use a thermometer, how do you use it to check the food?	Place on top of food	5 (0.6)
	Place thermometer on side of food	2 (0.3)
	Place thermometer in the centre of the thickest part of the food	109 (13.9)
	Place thermometer on the side of the pot	3 (0.4)
	I don’t use a thermometer	668 (84.9)
If you do not use a thermometer, what is the main reason for not using it?	I know the food is cooked by checking its visual appearance	356 (53.3)
	Troublesome to use it	102 (15.3)
	Other people don’t use it	31 (4.6)
	Don’t know how to use it	73 (10.9)
	There is no need for a thermometer–I’ve not had any food poisoning problems	49 (7.3)
	It can be a source of contamination	57 (8.5)

Table 2 shows the result of food safety knowledge, proportion of correct answers and standard loading of items. All standard loadings were > 0.40. More than 80% of the respondents answered half of the questions correctly. In K4 however, only 3.2% of the respondents knew that raw chicken should not be washed prior to preparation. Tables 3 and 4 present the results of food safety attitude and practices scale. Similarly, all standard loadings were higher than 0.40. Respondents mostly demonstrated positive attitude and safe food practices. The overall mean score for A2 was 2.92±1.29 and represented a general disagreement among the respondents in their attitude when sneezing or coughing (Table 2). Although respondents did not agree with the use of thermometer (2.90±1.08) (Table 3), most relied on visual appearance (4.00±1.18) to determine if the food is thoroughly cooked, e.g. fish should be flaky and opaque, or egg yolk and egg white should be firm (Table 4).

**Table 2:** Food safety knowledge and frequency of correct answers.

Item	Description	Correct answers Frequency (%)	Standard loadings
K1	Hands should be washed before meal preparation to prevent food poisoning	Yes 778 (98.9)	0.74
K2	Diarrhoea can be transmitted by consuming contaminated food	Yes 748 (95.0)	0.66
K3	Pets are allowed into the kitchen area	No 666 (84.6)	0.48
K4	Raw chicken should be washed before preparation	No 25 (3.2)	0.60
K5	If cooking meat and poultry, the juices should be clear and not pink when cooked	Yes 647 (82.2)	0.47
K6	Runny eggs are safe to eat	No 413 (52.5)	0.62
K7	Separate equipment such as chopping boards and utensils are used for raw meat and cooked food	Yes 632 (80.3)	0.53
K8	Raw meat can be stored anywhere in the refrigerator as long as it’s chilled	No 661 (84.0)	0.55
K9	Food preparation utensils can be washed with pipe water only	No 435 (55.3)	0.64
K10	Frozen food is thawed at room temperature	No 128 (16.3)	0.52
K11	Cooked food should be served hot (more than 60°C)	Yes 470 (59.7)	0.54
K12	Leftover food can be stored at room temperature to be eaten at the next meal	No 565 (71.8)	0.49

**Table 3:** Mean scores of items in attitude towards food safety (1: Strongly disagree to 5: Strongly agree).

Items	Description	Mean	Standard deviation	Standard loadings
A1	Washing hands with soap can prevent food poisoning	3.91	1.41	0.91
A2	When coughing / sneezing, we should cough/sneeze into our elbow if we do not have a tissue close by	2.92	1.29	0.53
A3	Hand injuries or cuts are covered to prevent cross contamination of food	3.89	1.39	0.91
A4	Fruits and vegetables (e.g. *ulam*) are washed before eating	4.14	1.46	0.94
A5	I do not use damaged or cracked eggs	3.97	1.47	0.87
A6	If I use a thermometer, I will clean it with water and soap each time after using	3.62	1.35	0.82
A7	Raw meat is stored at the bottom of the refrigerator shelf	2.37	1.39	0.54
A8	If there is only one chopping board, it should be washed after using it to prepare raw meat / poultry / seafood	3.92	1.44	0.90
A9	It is adequate to use one kitchen towel for all cleaning and drying purposes	1.89	1.10	0.79
A10	Leftover food is kept at room temperature, so I don’t have to reheat it	1.72	0.95	0.80
A11	Thermometer should be used to check if a food is thoroughly cooked	2.90	1.08	0.52
A12	Frozen food is kept at room temperature to defrost	3.27	1.35	0.68

**Table 4:** Mean scores of items in food safety practices (1: Never to 5: Always).

Items	Description	Mean	Standard deviation	Standard loadings
P1	I wash my hands with soap after using the toilet	4.21	1.22	0.85
P2	I wash my hands if I sneezed or coughed into my hands while preparing food	4.14	1.27	0.83
P3	If I have a pet (e.g. cat or dog) it’s free to roam in the kitchen area	1.92	1.21	0.67
P4	When purchasing food, I select fresh and wholesome food	4.28	1.15	0.89
P5	I wash raw meat before cutting or preparing them	4.29	1.25	0.82
P6	I do not use food beyond its expiry date	4.10	1.33	0.77
P7	I clean food preparation areas and utensils after preparing raw meat / poultry / seafood	4.32	1.19	0.90
P8	I chopped vegetables using a separate or a clean chopping board	3.86	1.34	0.72
P9	I use the same kitchen towel to wipe kitchen surfaces and dry my hands	1.93	1.26	0.69
P10	I check if the food is cooked by tasting it	3.43	1.38	0.44
P11	I check if the food is cooked by visual appearance (e.g. fish should be opaque and flaky; egg yolk and white should be firm)	4.00	1.18	0.76
P12	Leftover food from lunch are kept at room temperature until the next meal (e.g. dinner)	2.72	2.29	0.47

**Fig 1:**
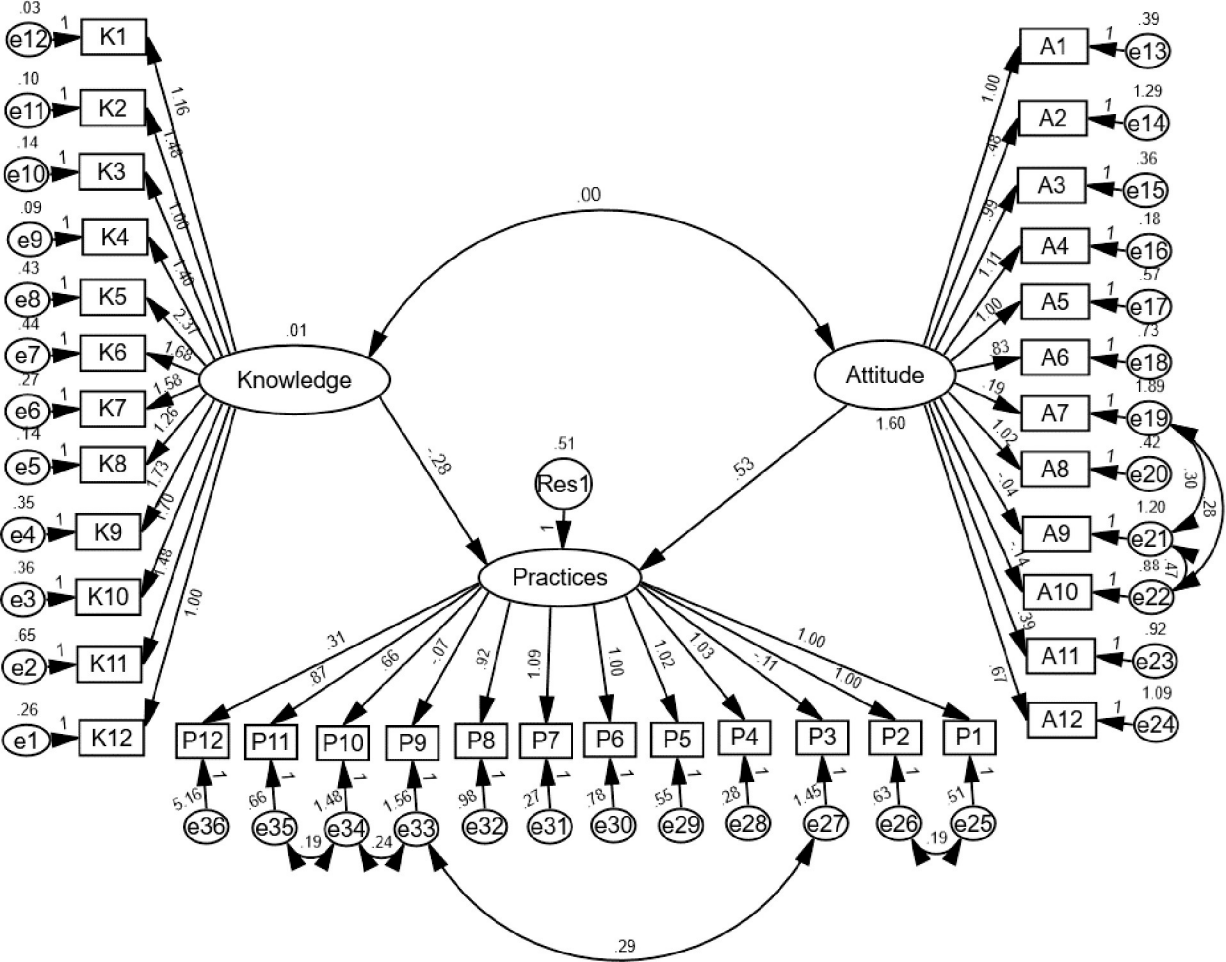
Model of food safety knowledge, attitude and practices. K: knowledge (K1-K12 = observed variables); A: Attitude; P: Practices; e: measurement error; Res: residual.

The Kaiser-Meyer-Olkin (KMO) measure of sampling values were 0.69, 0.94 and 0.93 for food safety knowledge, attitude and practices. According to [25], the KMO values should be more than 0.60, hence the criterion of validity is met. Table 5 shows the various goodness of fit indices in comparison with reported accepted values. Whilst evaluating the goodness of fit indices, it is recommended to use more than one indicator to evaluate model fit [20, 26]. Apart from Normed Fit Index (NFI) which measured slight below 0.90, all fit indices fulfil the accepted values. Both Comparative Fit Index (CFI) and Goodness of Fit Index (GFI) were estimated at 0.937 and 0.906 and indicates good fit. The Root Mean Square Error of Approximation (RMSEA) measured below 0.10 and was considered a good fit [27] while Root Mean Square Residual (RMR) was below 0.08, stipulating an acceptable fit [28]. Thus, the hypothesised model for food safety knowledge, attitude and practices had a good fit and is acceptable. The structural model between the variables of food safety knowledge, attitude and practices is shown in Fig 1.

**Table 5:** Goodness of fit indices.

Fit indices	Model value	Accepted value
ϰ^2^/df	2.335	< 3 [29]
Comparative Fit Index (CFI)	0.937	> 0.90 [28]
Goodness of Fit Index (GFI)	0.906	> 0.90 [30]
Root Mean Square Error of Approximation (RMSEA)	0.041	< 0.10 [27]
Normed Fit Index (NFI)	0.894	> 0.90 [31]
Root Mean Square Residual (RMR)	0.054	< 0.08 [28]

The direction and extent of relationships in the food safety knowledge and attitude and their direct effects on practices model is shown in Fig 1 and Table 6. Food safety knowledge has a negative and insignificant relationship (β1 = -0.284, p>0.05) with food safety practices hence rejecting hypothesis 1. The findings clearly indicate that food safety knowledge does not directly affect food safety practices. Based on the magnitude (i.e. β1 = -0.284, p>0.05) and direction (i.e. negative relationship) of the model, food safety practices will possibly decrease 0.28 unit with each unit increase in knowledge. The second hypothesis (H2) postulates that attitude directly affects food safety practices. H2 is sustained as the SEM demonstrates a positive and significant relationship between the two variables (β1 = 0.534, p<0.05). There is an insignificant relationship between food safety knowledge and attitude in this study (β1 = 0.005, p>0.05) thus H3 is rejected.

**Table 6:** Estimates of hypothesis paths food safety knowledge, attitude and practice.

Hypothesis	Paths	Estimate	Composite Reliability (C.R.)	P value
H1	Knowledge → Practice	-0.284	-0.765	0.444
H2	Attitude → Practice	0.534	19.47	0.000
H3	Knowledge <—> Attitude	0.005	0.837	0.402

## Discussion

This is the first study to report on thermometer usage among consumers in Malaysia. The findings support [32] who reported similar results i.e. 14% of at-home meal preparers use a food thermometer during a typical week. The main reason given by the respondents in this study was that they could rely on visual appearance to check if food is thoroughly cooked. This is in line with [33] recommendations to check that meat and fish are thoroughly cooked by making sure that there is no pink meat left, juices should run clear when the thickest part of the meat is pierced and fish should be opaque and flakes easily. However, a meat thermometer is used in modern kitchen to measure the internal temperature of cooked food [33]. Other main reasons given were: troublesome to use, don’t know how to use and it could be a source of contamination. The barriers to thermometer usage in this study echoes [34] who categorised the barriers into ‘belief that a thermometer is not necessary’ and ‘difficulty of selecting and using a thermometer’. [35] identified cooking habits and the influence of society and media as barriers to meat thermometer usage. Other previous studies in developing countries found up to 94% (n = 1393) consumers in Mainland China [36] and 93% (n = 1172) Lebanese [37] did not use thermometer to check food is cooked. [16] conducted a food safety training intervention study among two groups of food handlers in Malaysia and reported no changes in knowledge on temperature control even after a series of training. One of the main constraints to thermometer usage was their unavailability or lack of thermometers for food handlers [38].

Apart from K4 and A2 as discussed below, the empirical results in this study revealed that respondents have good level of food safety knowledge, positive attitude and self-reported practices in safe food handling. This agrees with [9] who reported Malaysian consumers exhibit high food safety knowledge and demonstrate positive food safety attitude and practices. Most respondents in this study reported washing raw chicken before cooking. This is similar to [39] where almost all respondents in Iraq and Egypt shared the practice of washing chicken in water before cooking. Some of the reported reasons for washing chicken before cooking were due to ‘food preparation practices taught at home’ and ‘to remove slime or to mask unwanted smell from raw chicken’ [39]. Food safety and public health organisations recommend not washing raw meat and poultry before preparation to reduce risk of foodborne illness due to cross contamination [40–42]. Washing raw poultry and meat could result in contamination of hands, sinks and counter-top surfaces [43].

The respondents in this study generally disagree with the etiquette of sneezing or coughing into the crook of the elbow if one does not have a tissue close by [44, 45]. In [46] and [47], the authors observed that most people coughed directly into their hands and did not immediately clean them. Sneezing and coughing can emit droplets of fluid and potentially infectious microorganisms and travel up to 7–8 meters [48, 49].

Fig 1 and Table 6 indicates that food safety knowledge does not translate into safe food handling practices. [18, 20, 50] also reported food safety and food allergen knowledge has a negative relationship with food safety practices. This could potentially be characterised by the optimistic bias (OB) phenomenon where consumers felt protected against food safety risks or ‘it won’t happen to me’ [51] or ‘he is worse than I am’ [52] perceptions. Meanwhile, food safety attitude was identified as an important factor in influencing food safety practices. A positive attitude indicates the level of motivation and care required to handle food safely. [12 and 14] similarly reported positive and significant relationship between food safety attitudes and practices among consumers in East Malaysia. Attitude represents beliefs and can serve as a crucial mediator between knowledge and practices [20]. An insignificant relationship between food safety knowledge and attitude reflect that both variables independently influenced practices. [6] found weak correlations between knowledge and hand hygiene attitudes among food handlers in Malaysia while [20] reported similar results among consumers. The current SEM model of food safety knowledge, attitude and practices (KAP) could be combined with the Theory of Planned Behaviour to understand how attitude, subjective norms and perceived behavioural control affect safe food handling practices. Specific KAP models on thermometer usage and handling of raw poultry and meat are worth studying in future studies.

### Limitations

This study is based on self-reported practices by a small number of respondents, hence could not be generalised to the whole population. A high percentage of the responses were made by those with tertiary education and this group of respondents are more likely to be aware of food safety issues and are motivated to carry out safe food handling practices. Correct thermometer usage is one of the main factors to consider in ensuring safe food. Although more than 50% of the respondents who don’t use a thermometer reported that they relied on visual cues, there is also the possibility of not owning a thermometer or popularity in using a thermometer in households that should be considered.

## Conclusion

The respondents in this study demonstrate good level of food safety knowledge and positive attitudes and self-reported practices. This is also the first study to report new empirical findings on thermometer usage among consumers in Malaysia. Respondents prefer to use visual cues to determine if food is cooked thoroughly while another main barrier was attributed to difficulty in using the device. Another key finding was almost all respondents would wash raw chicken prior to cooking. This could potentially increase risk of foodborne illnesses due to cross contamination. Respondents were also not likely to cough or sneeze into their elbows if tissue is unavailable, raising risk of cross contamination. The structural equation modelling showed a good fit on food safety knowledge, attitude and practices. Within SEM, although respondents are generally knowledgeable about food safety, this did not translate directly into food safety practices. However, attitude has been identified as a significant factor in influencing food safety practices. An insignificant relationship between knowledge and attitude suggests that the attributes affect food safety practices independently. Safe food handling practices at home play a critical role in protecting individuals and households, contributes to an overall improved social and quality of life and reduce burden on national health care.

## Supporting information

S1 Questionnaire Food safety knowledge, attitude and practices among consumers in Malaysia. (DOCX)

S1 Data (XLSX)
