# Supplementary material for: Structural equation modelling of food safety knowledge, attitude and practices among consumers in Malaysia
Source: PLoS One. 2020 Jul 8;15(7):e0235870. doi: 10.1371/journal.pone.0235870 (PMC7343170; doi:10.1371/journal.pone.0235870)
Supplement: S1 Questionnaire — (DOCX) [file pone.0235870.s001.docx]

**Questionnaire**

***Soal selidik***

**Food safety knowledge, attitude and practices among consumers in Malaysia**

***Pengetahuan, sikap dan amalan keselamatan makanan di kalangan pengguna di Malaysia***

| No. | English | *Malay* |
| --- | --- | --- |
|  | Questions | *Soalan* |
| 1 | Gender | *Jantina* |
|  | Male | *Lelaki* |
|  | Female | *Perempuan* |
| 2 | Age | *Umur* |
|  | 18-25 yrs | *18-25 tahun* |
|  | 26 -35 yrs | *26 -35 tahun* |
|  | 36 – 45 yrs | *36 – 45 tahun* |
|  | 46 – 55 yrs | *46 – 55 tahun* |
|  | 56 – 65 years | *56 – 65 tahun* |
|  | 65yrs and above | *66 tahun dan ke atas* |
| 3 | Education | *Tahap pendidikan* |
|  | Primary | *Tamat sekolah rendah* |
|  | Secondary | *Tamat sekolah Menengah* |
|  | Tertiary | *Tamat kolej / universiti* |
| 4 | Have you experienced food poisoning before? | *Pernahkah anda mengalami keracunan makanan sebelum ini?* |
|  | Yes | *Ya* |
|  | No | *Tidak* |
|  | Uncertain | *Tidak pasti* |
| 5 | What were the symptoms experienced? Please tick all that applies: | *Apakah simptom yang dialami? Sila tanda mana-mana yang berkenaan:* |
|  | Nausea | *Loya* |
|  | Vomiting | *Muntah* |
|  | Diarrhoea | *Cirit birit* |
|  | Stomach cramps | *Sakit perut* |
|  | Fever | *Demam* |
|  | Tired | *Letih* |
|  | Aches | *Rasa sakit* |
|  | Chills | *Menggigil* |
|  | Headaches | *Sakit kepala* |
|  | Loss of appetite | *Hilang selera makan* |
| 6 | Do you prepare your own / for family meals? | *Adakah anda menyediakan makanan untuk sendiri / keluarga anda?* |
|  | Yes | *Ya* |
|  | No | *Tidak* |
|  | Sometimes | *Kadang-kadang* |
| 7 | If you use a thermometer, how do you use it to check the food? | *Jika anda guna termometer, bagaimanakah anda menggunakannya untuk memeriksa makanan?* |
|  | Place thermometer on top of food | *Letakkan termometer di atas makanan* |
|  | Place thermometer on side of food | *Letakkan termometer di sebelah makanan* |
|  | Place thermometer in the centre of the thickest part of the food | *Letakkan termometer di bahagian tengah makanan yang paling tebal* |
|  | Place thermometer on the side of the pot | *Letakkan termometer di tepi periuk* |
|  | I don’t use a thermometer | *Saya tidak guna termometer* |
| 8 | If you do not use a thermometer, what is the main reason for not using it? | *Jika anda tidak guna termometer, apakah sebab utama anda tidak menggunakannya?* |
|  | I know the food is cooked by checking its visual appearance | *Saya tahu makanan tersebut sudah masak melalui penampilan visual.* |
|  | Troublesome to use it | *Leceh untuk menggunakannya* |
|  | Other people don’t use it | *Orang lain tidak menggunakannya* |
|  | Don’t know how to use it | *Tidak tahu bagaimana menggunakannya* |
|  | There is no need for a thermometer – I’ve not had any food poisoning problems | *Tidak perlu termometer - Saya tidak mengalami masalah keracunan makanan* |
|  | It can be a source of contamination | *Ia boleh menjadi sumber pencemaran* |
|  | Others, please state | *Lain-lain, sila nyatakan* |

## We would like to understand consumer’s awareness of food safety. Please select the most appropriate response for each statement:

## *Kami ingin memahami kesedaran pengguna berkaitan dengan keselamatan makanan. Sila pilih kenyataan yang paling sesuai:*

| 9 | Food safety knowledge | *Pengetahuan Keselamatan Makanan* | Yes  *Ya* | No  *Tidak* | Uncertain  *Tidak pasti* |
| --- | --- | --- | --- | --- | --- |
| a | Hands should be washed before meal preparation to prevent food poisoning | *Tangan perlu dibasuh sebelum penyediaan makanan untuk mencegah keracunan makanan* |  |  |  |
| b | Diarrhoea can be transmitted by consuming contaminated food | *Cirit birit boleh disebarkan dengan memakan makanan yang tercemar.* |  |  |  |
| c | Pets are allowed into the kitchen area | *Haiwan peliharaan dibenarkan masuk ke kawasan dapur* |  |  |  |
| d | Raw chicken should be washed before preparation | *ayam mentah perlu dibasuh sebelum penyediaan* |  |  |  |
| e | If cooking meat and poultry, the juices should be clear and not pink when cooked | *Jika memasak daging dan ayam, jus daging/ayam mestilah jelas dan tidak berwarna merah jambu setelah dimasak* |  |  |  |
| f | Runny eggs are safe to eat | *Telur yang cair adalah selamat untuk dimakan* |  |  |  |
| g | Separate equipment such as chopping boards and utensils are used for raw meat and cooked food | *Peralatan berasingan seperti papan memotong dan perkakas digunakan untuk daging mentah dan makanan yang telah masak* |  |  |  |
| h | Raw meat can be stored anywhere in the refrigerator as long as it’s chilled | *Daging mentah boleh disimpan di mana-mana sahaja dalam peti sejuk asalkan ia sejuk* |  |  |  |
| i | Food preparation utensils can be washed with pipe water only | *perkakas dapur penyediaan makanan boleh dicuci dengan air paip sahaja* |  |  |  |
| j | Frozen food is thawed at room temperature | *makanan beku dicairkan pada suhu bilik* |  |  |  |
| k | Cooked food should be served hot (more than 60ºC) | *Makanan yang telah dimasak harus dihidangkan panas (lebih daripada 60ºC)* |  |  |  |
| l | Leftover food can be stored at room temperature to be eaten at the next meal | *Makanan yang lebih boleh disimpan pada suhu bilik untuk dimakan pada hidangan seterusnya* |  |  |  |

| 10 | Food safety attitude | *Sikap keselamatan makanan* | Strongly disagree  *Sangat tidak setuju* | Disagree  *Tidak setuju* | Uncertain  *Tidak pasti* | Agree  *Setuju* | Strongly Agree  *Sangat setuju* |
| --- | --- | --- | --- | --- | --- | --- | --- |
| a | Washing hands with soap can prevent food poisoning | *Mencuci tangan dengan sabun boleh mencegah keracunan makanan* |  |  |  |  |  |
| b | When coughing / sneezing, we should cough/sneeze into our elbow | *Apabila batuk / bersin, kita harus batuk / bersin ke siku kita* |  |  |  |  |  |
| c | Hand injuries or cuts are covered to prevent cross contamination of food | *Tangan yang cedera / luka dilindungi untuk mengelakkan pencemaran makanan* |  |  |  |  |  |
| d | Fruits and vegetables (e.g. *ulam*) are washed before eating | *Buah-buahan dan sayur-sayuran (seperti ulam) dicuci sebelum makan* |  |  |  |  |  |
| e | I do not use damaged or cracked eggs | *Saya tidak menggunakan telur yang rosak atau retak* |  |  |  |  |  |
| f | If I use a thermometer, I will clean it with water and soap each time after using | *Jika saya menggunakan termometer, saya akan membersihkannya dengan air dan sabun setiap kali selepas menggunakannya* |  |  |  |  |  |
| g | Raw meat is stored at the bottom of the refrigerator shelf | *Daging mentah disimpan di bahagian bawah rak peti sejuk* |  |  |  |  |  |
| h | If there is only one chopping board, it should be washed after using it to prepare raw meat / poultry / seafood | *Jika hanya ada satu papan pemotong, ia perlu dibasuh setiap kali selepas menggunakannya untuk menyediakan daging mentah / ayam / makanan laut* |  |  |  |  |  |
| i | It is adequate to use one kitchen towel for all cleaning and drying purposes | *Ia adalah memadai untuk menggunakan satu tuala dapur untuk semua keperluan pembersihan dan pengeringan* |  |  |  |  |  |
| j | Leftover food is kept at room temperature so I don’t have to reheat it | *Sisa makanan disimpan pada suhu bilik jadi saya tidak perlu memanaskannya semula* |  |  |  |  |  |
| k | Thermometer should be used to check if a food is thoroughly cooked | *Termometer harus digunakan untuk memeriksa sama ada makanan sudah masak* |  |  |  |  |  |
| l | Frozen food is kept at room temperature to defrost | *Makanan sejuk beku disimpan pada suhu bilik untuk mencair* |  |  |  |  |  |

| 11 | Food safety practices | *Amalan keselamatan makanan* | Never  *Tidak pernah* | Seldom  *Jarang* | Sometimes  *Kadang-kadang* | Often  *Seringkali* | Always  *Sentiasa* |
| --- | --- | --- | --- | --- | --- | --- | --- |
| a | I wash my hands with soap after using the toilet | *Saya membasuh tangan dengan sabun selepas menggunakan tandas* |  |  |  |  |  |
| b | I wash my hands if I sneezed or coughed into my hands while preparing food | *Saya mencuci tangan saya jika saya bersin atau batuk ke tangan saya semasa menyediakan makanan* |  |  |  |  |  |
| c | If I have a pet (e.g. cat or dog) it’s free to roam in the kitchen area | *Jika saya mempunyai haiwan kesayangan (seperti kucing atau anjing) itu bebas berkeliaran di kawasan dapur* |  |  |  |  |  |
| d | When purchasing food, I select fresh and wholesome food | *Apabila membeli makanan, saya memilih makanan yang segar dan berkhasiat* |  |  |  |  |  |
| e | I wash raw meat before cutting or preparing them | *Saya mencuci daging mentah sebelum memotong atau menyediakannya* |  |  |  |  |  |
| f | I do not use food beyond its expiry date | *Saya tidak menggunakan makanan yang telah tamat tarikh luputnya* |  |  |  |  |  |
| g | I clean food preparation areas and utensils after preparing raw meat / poultry / seafood | *Saya membersihkan kawasan penyediaan makanan dan peralatan selepas menyediakan mentah daging / ayam / makanan laut* |  |  |  |  |  |
| h | I chopped vegetables using a separate or a clean chopping board | *Saya cincang sayur-sayuran dengan menggunakan papan pemotong yang berasingan atau yang bersih* |  |  |  |  |  |
| i | I use the same kitchen towel to wipe kitchen surfaces and dry my hands | *Saya menggunakan tuala dapur yang sama untuk mengelap permukaan dapur dan untuk mengeringkan tangan saya* |  |  |  |  |  |
| j | I check if the food is cooked by tasting it | *Saya memeriksa sama ada makanan sudah masak dengan mencuba rasanya* |  |  |  |  |  |
| k | I check if the food is cooked by visual appearance (e.g. fish should be opaque and flaky; egg yolk and white should be firm) | *Saya memeriksa sama ada makanan sudah masak melalui penampilan visual (contoh: ikan harus menjadi legap dan bersepih; telur kuning dan putih harus menjadi kukuh)* |  |  |  |  |  |
| l | Leftover food from lunch are kept at room temperature until the next meal (e.g. dinner) | *Sisa makanan dari makan tengahari disimpan pada suhu bilik sehingga hidangan seterusnya (contoh: makan malam)* |  |  |  |  |  |
